# Enhancing the dataset of CycleGAN-M and YOLOv8s-KEF for identifying apple leaf diseases

**DOI:** 10.1371/journal.pone.0321770

**Published:** 2025-05-30

**Authors:** Lijun Gao, Hongxin Wu, Yunsheng Sheng, Kunlin Liu, Huanhuan Wu, Xuedong Zhang

**Affiliations:** 1 College of Information Engineering, Tarim University, City of Aral, China; 2 Key Laboratory of Tarim Oasis Agriculture, Ministry of Education, City of Aral, China; University of Sharjah, UNITED ARAB EMIRATES

## Abstract

Accurate diagnosis of apple diseases is vital for tree health, yield improvement, and minimizing economic losses. This study introduces a deep learning-based model to tackle issues like limited datasets, small sample sizes, and low recognition accuracy in detecting apple leaf diseases. The approach begins with enhancing the CycleGAN-M network using a multi-scale attention mechanism to generate synthetic samples, improving model robustness and generalization by mitigating imbalances in disease-type representation. Next, an improved YOLOv8s-KEF model is introduced to overcome limitations in feature extraction, particularly for small lesions and complex textures in natural environments. The model’s backbone replaces the standard C2f structure with C2f-KanConv, significantly enhancing disease recognition capabilities. Additionally, we optimize the detection head with Efficient Multi-Scale Convolution (EMS-Conv), improving the model’s ability to detect small targets while maintaining robustness and generalization across diverse disease types and conditions. Incorporating Focal-EIoU further reduces missed and false detections, enhancing overall accuracy. The experiment results demonstrate that the YOLOv8s-KEF model achieves 95.0% in accuracy, 93.1% in recall, 95.8% in precision, and an F1-score of 94.5%. Compared to the original YOLOv8s model, the proposed model improves accuracy by 7.2%, precision by 6.5%, and F1-score by 5.0%, with only a modest 6MB increase in model size. Furthermore, compared to Faster RCNN, ResNet50, SSD, YOLOv3-tiny, YOLOv6, YOLOv9s, and YOLOv10m, our model demonstrates substantial improvements, with up to 30.2% higher precision and 18.0% greater accuracy. This study used CycleGAN-M and YOLOv8s-KEF methods to enhance the detection capability of apple leaf diseases.

## 1. Introduction

Apples are cultivated worldwide and are among the most common fruits due to their high nutritional value and significant health benefits, making them one of the most productive crops globally [1]. According to the China Apple Industry Association (http://www.chinaapple.org.cn), China dominates global apple production and consumption, contributing to over half of the world’s apple-growing acreage, thereby playing a decisive role in the global apple industry [2]. As a major economic crop in China, apples experience an average loss rate of 12%–16% due to diseases. Common apple diseases include rust, scab, leaf spot, and powdery mildew [3,4]. Traditional disease identification methods primarily rely on farmers’ experience, visual inspections, and chemical treatments, leading to challenges such as high costs, reliance on subjective judgment, and low efficiency. Due to the wide variety of diseases and the large number of leaves, this approach is prone to errors and can result in disease spread if misjudged [5]. Therefore, timely and accurate detection of apple leaf diseases is critical to ensuring the health of the apple industry and has become a significant research focus in agricultural informatics.

Deep learning has found broad use in agriculture for identifying and classifying plant diseases. Various neural network models, including AlexNet [6], VGG [7], GoogleNet [8], ResNet [9], DenseNet [10], and MobileNet [11], have demonstrated remarkable success in disease identification. Pushpa et al. [12] proposed an AlexNet-based model that achieved 96.7% accuracy, providing an effective tool for early disease identification in crops like corn, rice, and tomatoes, thus improving agricultural efficiency. Paymode et al. [13] used the VGG model to classify healthy and diseased leaves of grapes and tomatoes, achieving accuracy rates of 98.40% and 95.71%, respectively. Ferentinos et al. [14] employed a convolutional neural network (CNN) architecture to evaluate an automatic plant disease detection system. The system analyzed images of healthy and diseased plant leaves, detecting 25 plant species and 58 different diseases, with a classification success rate of 99.53% on the test set. Khan et al. [1] developed a deep-learning approach to apple disease detection, using a dataset of about 9,000 high-quality RGB images. Their lightweight classification and detection models achieved 88% accuracy and a mAP_@0.5_ of 42%, effectively addressing the challenges of identifying and locating apple leaf diseases. These studies demonstrate that deep learning technology enables quick and accurate crop health monitoring, helping farmers take timely measures to prevent disease spread and ensure optimal crop growth and yield.

To refine the functionality of network models, large amounts of data are required for training. Traditional data augmentation techniques, such as random flipping [15], shearing [16], rotation [17], Gaussian noise [18], and translation [19], generate new images with semantic similarity to the original ones, but they do not fundamentally solve the issue of dataset diversity. With the advent of generative adversarial networks (GANs) [20,21], data augmentation has advanced significantly. Unlike traditional methods, GANs can simulate the statistical properties of natural scenes and generate large amounts of realistic image data without the need for manual annotation, thus alleviating the issue of insufficient training data. Many researchers have applied GANs in various fields, including noise removal [22], text-to-image conversion [23], image super-resolution [24], and style transfer [25]. Studies have shown that augmenting datasets with synthetic samples generated by GANs can address data scarcity and enhance model performance. Wang et al. [26] used a WGAN to generate tomato disease images and employed the MCA-MobileNet network for disease recognition. Their experiments demonstrated that GAN-based data augmentation not only increased sample size but also improved classification accuracy. Zhou et al. [27] introduced a fine-grained GAN to enhance grape leaf training data in local spot areas. By mixing generated and original images, their model achieved 96.27% accuracy on ResNet-50, which is crucial for handling rare diseases or limited training samples. Chen et al. [28] combined CycleGAN with YOLOv4 for multi-angle optical inspection and automatic image annotation, improving the efficiency and accuracy of surface defect detection in golden pineapples. Their method achieved 84.86% accuracy at 6.41 frames per second, reducing reliance on manual inspection and providing an effective solution for automated agricultural defect detection. Zhang et al. [29] proposed a high-quality image enhancement (HQIA) method using an improved dual WGAN-GP to generate high-resolution images of rice leaf diseases. The ResNet-18VGG11 model, trained with this data, achieved a 4.57% and 4.1% improvement in accuracy, respectively. While existing studies highlight GANs’ potential for generating synthetic agricultural images to augment datasets, challenges remain, such as insufficient sample diversity and lower accuracy in complex environments. The quality and fidelity of synthetic data still need improvement.

Although these object detection algorithms have achieved remarkable success in detecting fruit tree diseases, certain limitations still exist. Detection networks are typically trained using sample data collected under controlled conditions, which may ignore unrestricted environmental factors such as changes in lighting and shadows, leaf reflections, and background interference from similar objects [30]. Insufficient consideration of these factors may limit the generalization and robustness of detection networks in practical applications. In addition, the varying sizes of lesions in fruit tree diseases pose significant challenges for detection models in accurately identifying them across different scales [31,32]. This study addresses these issues by proposing a method for recognizing apple leaf diseases in images based on an improved CycleGAN-M and YOLOv8s-KEF. The primary contributions of this study are as follows:

(1) An apple leaf disease detection dataset was constructed by extracting original images from a publicly available apple leaf disease dataset. These images were annotated and converted to the YOLO dataset format. The dataset was then expanded with additional samples, and the newly augmented dataset was used for apple leaf disease detection tasks.
(2) An improved CycleGAN-M is proposed to generate synthetic apple leaf disease data. A multi-scale attention mechanism is introduced into the generator, allowing the model to adaptively weight leaf image features at different spatial scales. This enables more accurate capture of structural and textural information in lesion areas. As a result, the model improves its ability to extract features of apple leaf disease spots, enhances the quality of generated images, and addresses issues related to the limited amount of disease data and the imbalance in disease spot categories.
(3) An improved YOLOv8s-KEF model for apple disease detection was proposed. The C2f-KanConv module was introduced to replace the original C2f structure in the backbone. Additionally, EMS-Conv was utilized to optimize the detection head structure, and Focal-EIoU was applied as the loss function for bounding box regression. These improvements significantly enhanced the model’s detection performance.

## 2. Materials and methods

### 2.1. Data sources

**Fig 1:**
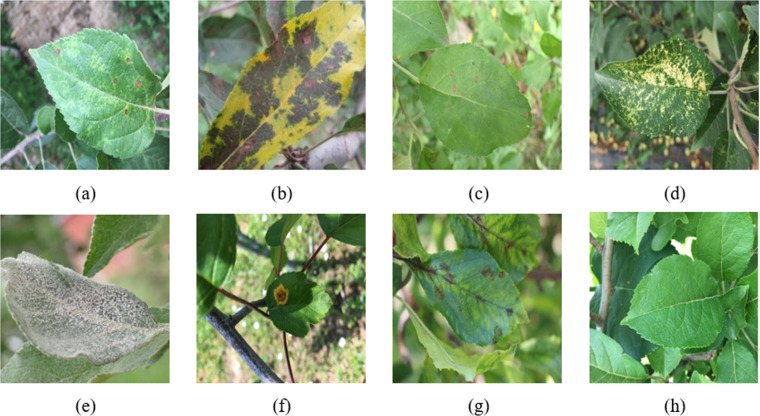
Partial images of apple leaf diseases: (a) Alternaria leaf spot; (b) Brown spot; (c) Frogeye leaf spot; (d) Mosaic; (e) Powdery mildew; (f) Rust; (g) Scab; (h) Health.

Insufficient data and low regional representativeness are significant challenges that hinder the performance of predictive models [33]. The dataset utilized in this research is the publicly available Apple Leaf Diseases dataset, sourced from the official Kaggle platform (https://www.kaggle.com). It covers healthy leaves and various common apple leaf diseases, such as leaf spots, brown spots, eye spots, grey spots, mosaic, powdery mildew, rust, and Scab. Fig 1 displays sample images depicting various apple leaf diseases.

**Fig 2:**
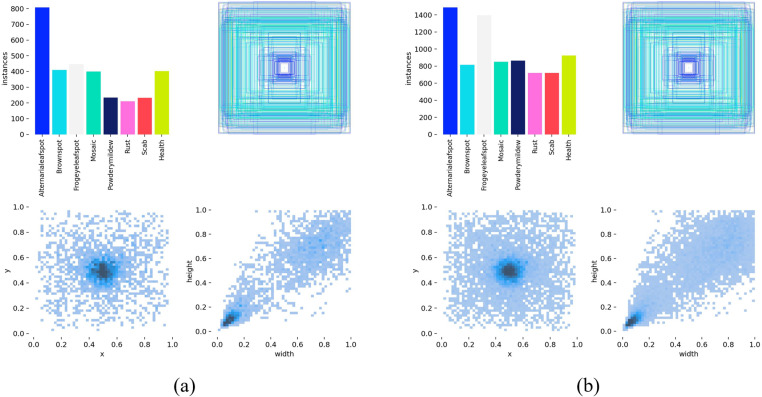
Visualization of Apple Leaf Disease Data Labels: (a) The length and width distribution of the original dataset labels; (b) The label length and width distribution of the enhanced dataset.

The study focused on seven prevalent apple leaf diseases, along with healthy leaves, creating a dataset of 3,663 images to improve the model’s robustness and generalization. This comprehensive collection comprises 417 images of leaf spot disease, 411 images depicting brown spot disease, and 481 images associated with frog-eye leaf spot disease. Furthermore, the dataset includes 371 images of mosaic disease, 494 images representing powdery mildew, and 479 images of rust disease. Lastly, it features 494 images of scab disease, along with 516 images of healthy leaves, providing a diverse set for analysis and model training. All images are stored in PNG format with a standardized resolution of 640×640 pixels. The statistics of the apple leaf disease dataset before and following data augmentation are presented in Table 1, while the distribution of the label lengths and widths in the dataset for apple leaf diseases is illustrated in Fig 2.

**Table 1:** Statistical comparison of the apple leaf disease dataset before and after data.

Number	Types	Number of original images (pieces)	Number of annotation boxes (pieces)	Number of images after data enhancement (pieces)	Number of annotation boxes after enhancement (pieces)
1	Alternaria leaf spots	417	800	937	1459
2	Brown spots	411	411	801	1361
3	Frogeye leaf spots	481	437	817	1383
4	Mosaic	371	418	891	1425
5	Powdery mildew	494	494	1001	1501
6	Rust	479	561	950	1425
7	Scab	494	536	838	1340
8	Health	516	613	1010	1313
9	Total	3663	4270	7245	11207

### 2.2. Image enhancement through CycleGAN deep learning techniques

**Fig 3:**
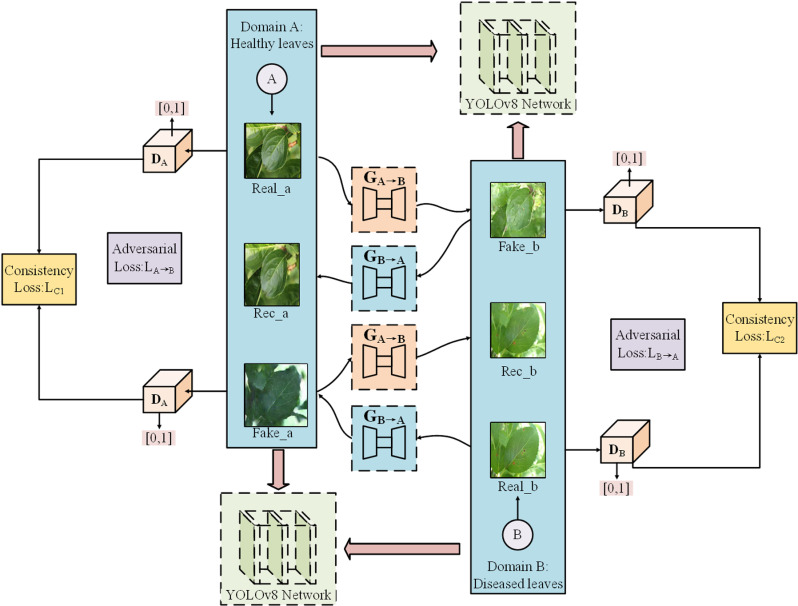
The principle of cycle-consistent generative adversarial networks.

The Cycle-Consistent Generative Adversarial Network (CycleGAN) is a neural network model utilizing convolutional layers designed for translating images from one domain to another, introduced by Zhu et al. [34]. Unlike the GAN introduced by Goodfellow and his team, CycleGAN [35,36] can perform mappings between two image domains using unpaired training data, eliminating the need for paired datasets. The architecture of CycleGAN, as illustrated in Fig 3, includes two generators (G_A→B_ and G_B→A_) and two discriminators (D_A_ and D_B_). Generator G_A→B_ translates images from domain A to domain B, while generator G_B→A_ performs the reverse translation from domain B to domain A. Discriminators D_A_ and D_B_ distinguish real images from generated ones in each domain. CycleGAN utilizes adversarial loss to prompt the generators to generate realistic images. Additionally, it incorporates cycle consistency loss, which ensures that the images can be transformed back to their original domain. This dual approach effectively maintains consistency between the input and output domains, enhancing the quality of the generated images.

CycleGAN comprises two pairs of generator-discriminator networks [37] for converting images from the source domain to the target domain (G_A→B_) and vice versa (G_B→A_). Its key innovation lies in the use of cycle consistency loss [38], which ensures that after two transformations, the image closely resembles its original form. Adversarial loss guides the generation of realistic images, as defined in Equation (1), while cycle consistency loss preserves the content of the images by minimizing reconstruction error, as defined in Equation (2).


$$L_{\text{GAN}}\left(\text{G},{\text{D}}_{\text{Y}},\text{X},\text{Y}\right)={\text{E}}_{\text{y}\sim \text{Pdata}\left(\text{y}\right)}\left[{\text{logD}}_{\text{Y}}\left(\text{y}\right)\right]+{\text{E}}_{\text{x}\sim \text{Pdata}\left(\text{x}\right)}\left[\text{log}\left(1-{\text{D}}_{\text{Y}}\left(\text{G}\left(\text{X}\right)\right)\right)\right]$$ (1)

$$L_{\text{cyc}}\left(\text{G},\text{F}\right)={\text{E}}_{\text{y}\sim \text{Pdata}\left(\text{x}\right)}\left[∥\text{F}(\text{G}\left(\text{x}\right){∥}_{1}\right]+{\text{E}}_{\text{y}\sim \text{Pdata}\left(\text{y}\right)}\left[∥\text{G}(\text{F}\left(\text{y}\right)-\text{y}{∥}_{1}\right]$$ (2)

The total loss of the generator is the combination of these two losses, as shown in Equation (3).


$$L_{\text{total}}\left(\text{G},\text{F},{\text{D}}_{\text{X}},{\text{D}}_{\text{Y}}\right)=L_{\text{GAN}}\left(\text{G},{\text{D}}_{\text{Y}},\text{X},\text{Y}\right)+L_{\text{GAN}}\left(\text{F},{\text{D}}_{\text{X}},\text{Y},\text{X}\right)+ℷL_{\text{GAN}}\left(\text{G},\text{F}\right)$$ (3)

#### 2.2.1 Generator of CycleGAN.

The CycleGAN generator comprises an encoder, a residual block, and a decoder [39]. The encoder extracts feature from the input image using a series of convolutional layers. The residual block maintains consistency between the input and output while learning complex features through two convolutional layers and skip connections. The decoder progressively restores the spatial information of the image via deconvolution layers, ultimately generating an image in the target domain. The design and parameter settings of each layer collectively ensure high-quality image conversion. Table 2 presents the network parameters of the CycleGAN generator.

**Table 2:** Generator network parameters.

Layers	Name	Input channel	Convolution kernel size	Padding	Output channel	Activation Function	Stride
1	Conv1	3	7×7	3	64	ReLU	1
2	Conv2	64	3×3	1	128	ReLU	2
3	Conv3	128	3×3	1	256	ReLU	2
4	ResBlock1_Conv1	256	3×3	1	256	ReLU	1
5	ResBlock1_Conv2	256	3×3	1	256	–	1
6	ResBlock2_Conv1	256	3×3	1	256	ReLU	1
7	ResBlock2_Conv2	256	3×3	1	256	–	1
8	ResBlock3_Conv1	256	3×3	1	256	ReLU	1
9	ResBlock3_Conv2	256	3×3	1	256	–	1
10	ResBlock4_Conv1	256	3×3	1	256	ReLU	1
11	ResBlock4_Conv2	256	3×3	1	256	–	1
12	ResBlock5_Conv1	256	3×3	1	256	ReLU	1
13	ResBlock5_Conv2	256	3×3	1	256	–	1
14	ResBlock6_Conv1	256	3×3	1	256	ReLU	1
15	ResBlock6_Conv2	256	3×3	1	256	–	1
16	Deconv1	256	3×3	1	128	ReLU	2
17	Deconv2	128	3×3	1	64	ReLU	2
18	Deconv3	64	7×7	3	3	Tanh	1

#### 2.2.2 Discriminator of CycleGAN.

The CycleGAN discriminator utilizes the PatchGAN architecture [40], which employs multiple convolutional layers to retrieve local features from the input image, distinguishing between real and generated images. Table 3 details the specifications of the discriminator network. The input to the network is an RGB image consisting of three channels, utilizing a convolutional kernel dimension of 4x4 with a stride of 2. This configuration gradually decreases the size of the feature map. The LeakyReLU activation function is used throughout the network, while the final layer applies the Sigmoid activation function, producing a value between 0 and 1 to indicate the likelihood that the input image is generated.

**Table 3:** Discriminator network parameters.

Layers	Name	Input channel	Convolution kernel size	Padding	Output channel	Activation Function	Stride
1	Conv1	64	4x4	1	64	LeakyReLU	2
2	Conv2	128	4x4	1	128	LeakyReLU	2
3	Conv3	256	4x4	1	256	LeakyReLU	2
4	Conv4	512	4x4	1	512	LeakyReLU	2
5	Conv5	1	4x4	1	1	Sigmoid	1

### 2.3. Introduction to the YOLOv8 model

**Fig 4:**
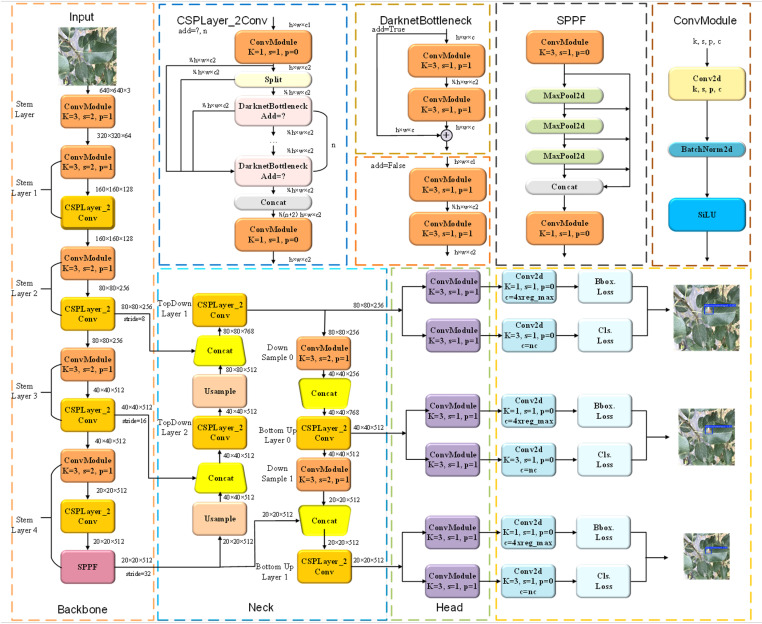
YOLOv8 model structure diagram.

The YOLO [41,42] series of algorithms offer advantages in terms of speed and real-time capability, rendering them highly efficient for object recognition tasks and well-suited for detecting crop diseases and pests. This research employs the YOLOv8 model for detecting diseases in apple leaves. The overall architecture of YOLOv8 is illustrated in Fig 4. YOLOv8 is a highly efficient single-stage object detection algorithm that includes a range of object detection models with varying network depth and width [43]. These models are classified into five versions, from smallest to largest: YOLOv8n, YOLOv8s, YOLOv8m, YOLOv8l, and YOLOv8x. The network structure of YOLOv8n comprises four key components: the input layer, backbone network, fusion layer (Neck), and detection head [44,45].

In the input layer, YOLOv8 utilizes Mosaic data augmentation to enrich the dataset, along with adjustable hyperparameters tailored to different model scales. For larger models, additional augmentation techniques such as MixUp and CopyPaste are applied to enhance model generalization and robustness. The backbone network is responsible for feature extraction and consists of several convolutional layers, C2f modules, and SPPF (Spatial Pyramid Pooling Fast). The convolutional layers handle the extraction and arrangement of feature maps, while the C2f module, inspired by the residual structure of YOLOv7 and C3 modules, efficiently captures gradient flow information and adjusts channel numbers based on model size to boost performance. The SPPF module merges features at different scales using spatial pyramid pooling operations.

The neck network integrates FPN (Feature Pyramid Network) and PAN (Path Aggregation Network) structures, enabling the effective fusion of the features obtained from the backbone and improving both multi-scale positioning accuracy and semantic representation. Lastly, the head network is accountable for identifying object types and their locations. It employs a decoupled head structure to distinguish between classification and detection, addressing the different objectives of object recognition and localization. Moreover, YOLOv8 employs an anchor-free detection approach, increasing detection speed.

## 3. Methods

### 3.1. Improvement of CycleGAN

**Fig 5:**
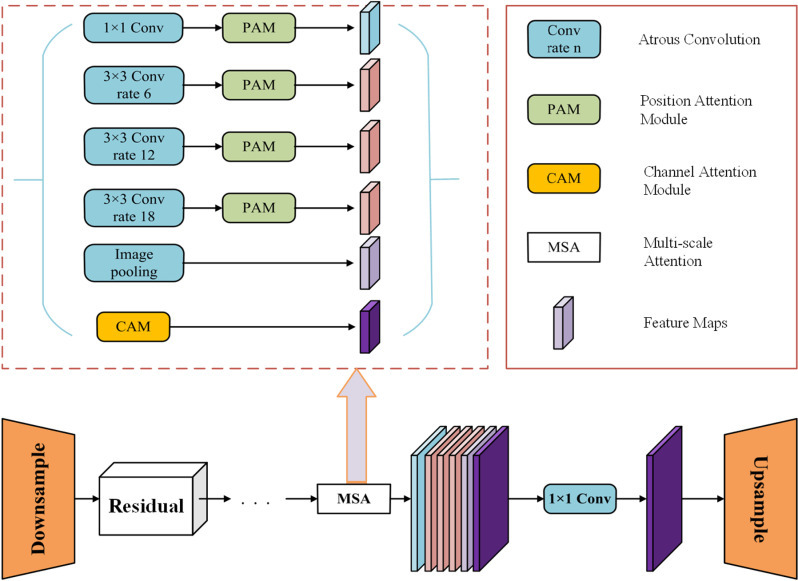
Multi-scale attention mechanism improves the CycleGAN generator structure.

Although the original CycleGAN network can extract features of diseases of apple leaves, the quality of the generated images remains suboptimal. This study proposes an enhanced CycleGAN-M model for apple leaf disease dataset augmentation, improving the generator’s feature extraction and image generation capabilities through the integration of a multi-scale attention (MSA) mechanism. The MSA mechanism captures disease features of apple leaves at different scales and effectively focuses on key regions, minimizing the interference of background noise on the generated images. By incorporating the MSA into the generator, CycleGAN-M not only enhances image clarity but also improves the model’s ability to recognize fine-grained disease characteristics, significantly increasing the diversity and authenticity of the generated images. The improved generator structure is illustrated in Fig 5.

The MSA comprises three parallel branches: the spatial attention component (PAM), the channel attention component (CAM), and the global average pooling layer (GAP). The spatial attention branch captures the interdependence between different spatial positions by extracting feature correlations across the spatial dimension. The channel attention branch focuses on the channel dimension, enhancing key feature representation by adjusting the weights of different feature channels. The global average pooling layer aggregates the global information of the entire image, further improving the model’s perception of the overall context.

For the input feature map F, this study extracts multi-scale spatial features using dilated convolutions with varying dilation rates r. Assuming that AstrousConvr represents a dilated convolution with dilation rate r, the multi-scale feature extraction is demonstrated in Equation (4). Dilated convolutions effectively expand the receptive field and capture long-range feature dependencies without increasing the number of parameters.


$${\text{F}}_{\text{multi}\_\text{scale}}=\left\{{\text{AtrousConv}}_{1}\left(\text{F}\right),{\text{AtrousConv}}_{6}\left(\text{F}\right),{\text{AtrousConv}}_{1}2\left(\text{F}\right),{\text{AtrousConv}}_{1}8\left(\text{F}\right)\right\}$$ (4)

After obtaining the feature map through multi-scale dilated convolution, the spatial attention is calculated. Initially, the input feature map is denoted as A and has dimensions of C×H×W, where C indicates the number of channels, H represents the height, and W denotes the width. Features B and C are obtained through two convolutional layers and then flattened into a format of C×N, where N equals H×W. The spatial attention matrix S is defined in Equation (5). The dimensions of both S and the feature are N×N, with each element Sij representing the correlation between the i-th position and the j-th position in the feature map. Next, spatial attention S is multiplied by the feature map D, and the input feature A is added to construct the residual structure. As shown in formula (6), α is a learnable scaling parameter.


$$S=softmax\left(B\cdot C^{T}\right)$$ (5)

$$\text{E}=\text{α}\left(S^{T}\cdot \text{D}\right)+\text{A}$$ (6)

For channel attention, we first aggregate information from the spatial dimensions through global average pooling (GAP) to compute the global features across the channels. Let the input feature map be denoted as F, with dimensions C×H×W. The calculation formula for channel attention is presented in Equation (7). Here, σ denotes the activation function, while W_1_ and W_2_ represent the learned weight matrices.


$${\text{M}}_{\text{c}}=\text{σ}\left({\text{W}}_{1}\cdot \text{GAP}\left(\text{F}\right)+{\text{W}}_{2}\right)$$ (7)

The global average pooling (GAP) layer further aggregates the spatial features into global contextual information, enhancing the capture of global features, as illustrated in Equation (8). The features extracted by the multi-scale attention mechanism are fused to retrieve the output disease image from the generator, as presented in Equation (9). Here, G represents the generator network, which generates realistic apple leaf disease images by integrating spatial attention, channel attention, and global features.


$${\text{F}}_{\text{GAP}}=\frac{1}{\text{H}\times \text{W}}\overset{\text{H}}{\underset{\text{i}=1}{\sum }}\overset{\text{W}}{\underset{\text{j}=1}{\sum }}\text{F}\left(\text{i},\text{j}\right)$$ (8)

$${\text{G}}_{\text{output}}=\left({\text{E}}_{\text{PAM}},{\text{M}}_{\text{CAM}},{\text{F}}_{\text{GAP}}\right)$$ (9)

### 3.2. Improvement of YOLOv8s structure

#### 3.2.1. C2f-KAN convolution module.

**Fig 6:**
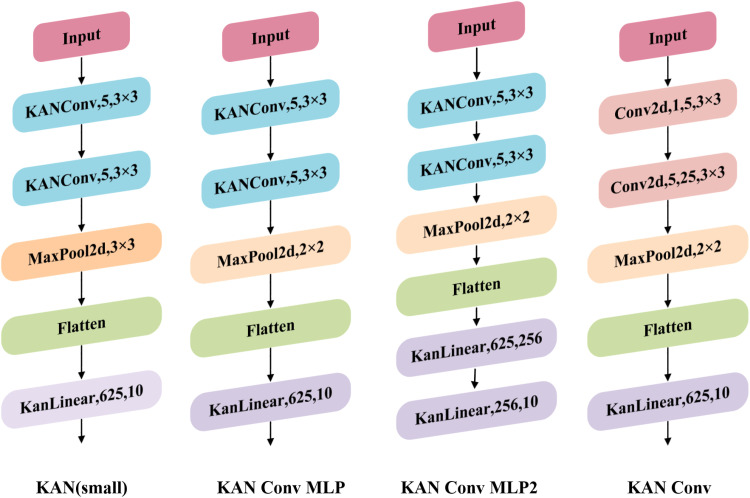
KAN convolution module structure.

The Kolmogorov-Arnold Network (KAN) [46,47] is an advanced convolutional neural network architecture able to dynamically modify the weights of the convolutional kernel to adapt to various input features. This dynamic adjustment improves the network’s capacity to effectively capture subtle characteristics of apple leaf diseases and improves disease identification. Compared to traditional convolutional neural networks, KAN effectively reduces redundant information when processing apple leaf images, thereby enhancing detection accuracy. The KANConv module is illustrated in Fig 6. Its design allows KAN to perform optimally when handling apple leaf images exhibiting multiple disease manifestations and complex backgrounds, contributing to improved detection accuracy while false positives and overlooked detections.

**Fig 7:**
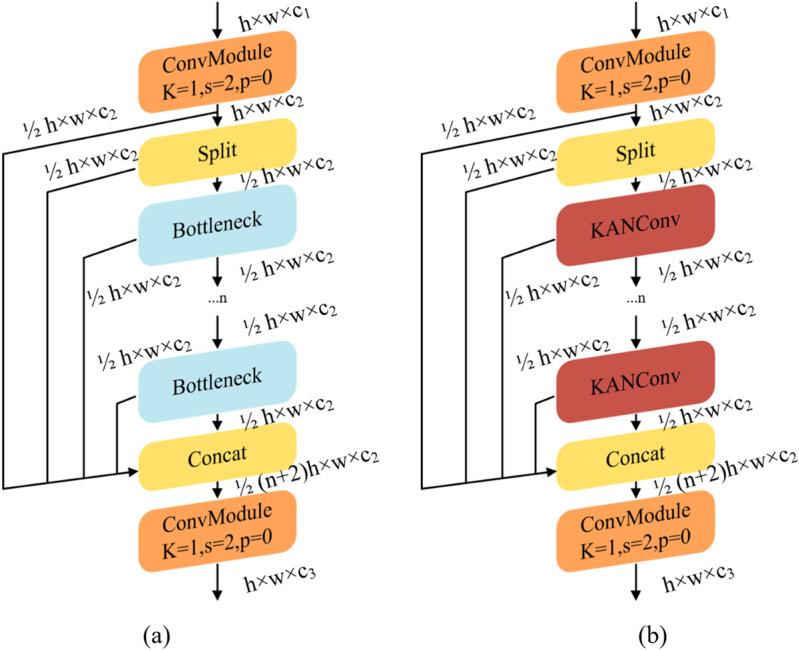
Module structure before and after improvement: (a) C2f module; (b) C2f-KAN module.

In the YOLOv8s model, the C2f module primarily extracts features through conventional convolution, and its feature learning effectiveness is crucial to the model’s overall performance. The extraction of apple leaf spot features in natural scenes may result in the retention of redundant information, which increases computational load and leads to issues such as false detections and missed detections. To tackle these issues, we propose replacing the C2f structure with C2f-KanConv to enhance apple leaf disease recognition. As illustrated in Fig 7, this structure incorporates KAN convolution within the C2f module, utilizing the kernel attention mechanism to dynamically adjust the convolution kernel weights. This enhancement improves feature extraction effectiveness and increases the accuracy of spot feature extraction.

#### 3.2.2. Detection head optimization.

**Fig 8:**
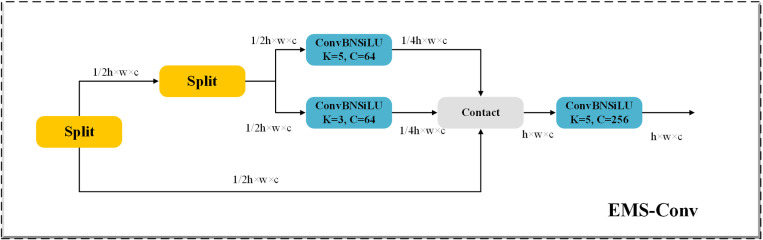
EMS-Conv module structure diagram.

In traditional convolutional neural networks (CNNs), the use of a single-scale convolution kernel limits the network’s ability to capture multi-scale features, thereby affecting its capacity to identify objects or scenes of varying sizes. In contrast, multi-scale convolution technology allows for a more comprehensive acquisition of information across different scales, enabling the extraction of richer features. Sun et al. [48] introduced an effective multi-scale convolution (EMS-Conv) module to facilitate multi-scale information fusion. The EMS-Conv module effectively captures target features at different scales through the parallel processing and dynamic fusion mechanisms of multi-scale convolution kernels, thereby improving the accuracy and efficiency of target detection while reducing computational complexity to some extent. Its working principle is illustrated in Fig 8. Initially, the input obtained through convolution is categorized into two groups according to the number of channels. The first group processes the source image to extract basic and unprocessed features. The second group further divides the unprocessed features into two parts, which are then convolved using 3 × 3 and 5 × 5 convolution kernels, respectively. Subsequently, the results of these operations are concatenated with the basic features extracted by the first group, forming independently connected features within each channel. Finally, after concatenation, a 1 × 1 convolutional layer is added to fuse the channel information, enhancing feature representation and achieving dimensionality reduction. This paper presents the idea of shared parameters and integrates it with the lightweight and efficient EMSPConv convolution to enhance the accuracy of detection for small objects in intricate backgrounds.

**Fig 9:**
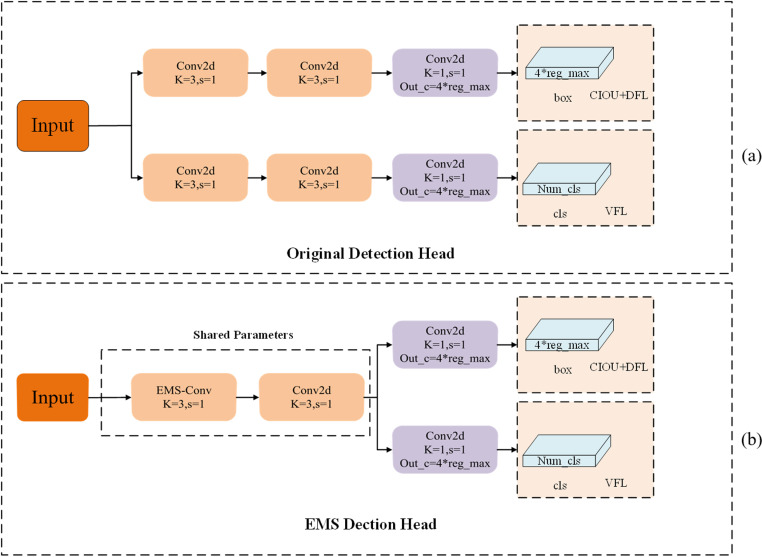
Comparison of detection heads: (a) the structure of the YOLOv8 detection head; (b) the Improved EMS detection head structure.

This approach effectively maintains high computational efficiency and model robustness. A comparison of the detection head before and after the improvements is illustrated in Fig 9.

#### 3.2.3. Focaler-EIOU loss function.

**Fig 10:**
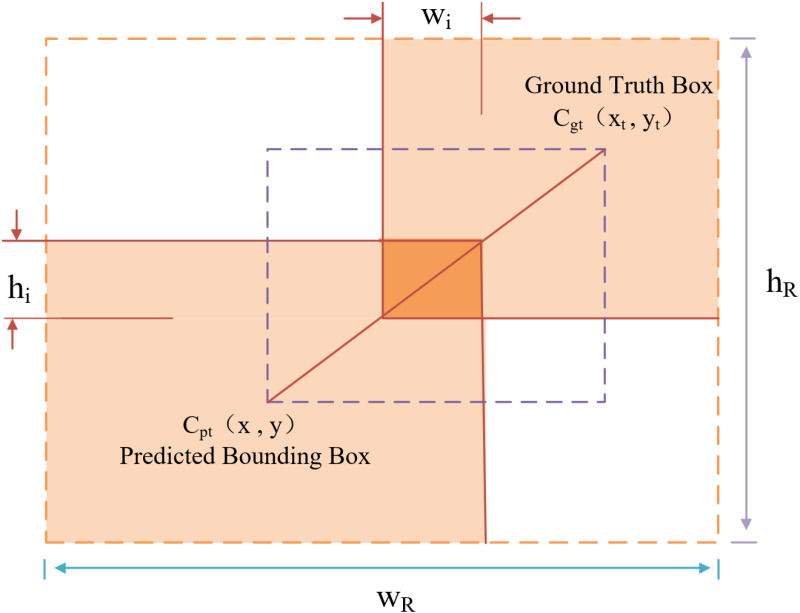
Cost calculation method for location information regression loss function based on IoU.

In target detection algorithms, accurate target localization is critical, and it is typically achieved through the bounding box regression module. The predicted bounding box is represented as B_P_= [x,y,w,h], and the true bounding box is represented as B_gt_= [x_gt_,y_gt_,w_gt_,h_gt_]. The Inter-Union (IOU) Loss is defined and illustrated in Fig 10.

In the natural environment, accurately identifying the location of lesions is a crucial aspect of apple leaf disease detection. To accelerate model convergence, enhance accuracy, and address the sample imbalance issue in bounding box regression tasks, we replaced the original loss function with the Focal-EIOU loss function [49,50]. This approach allows for a more comprehensive evaluation of overlap, distance, and aspect ratio between the predicted and actual bounding boxes, thereby improving the precision of bounding box regression. In the YOLOv8s network, the CIoU Loss function is typically used for bounding box regression. The calculation of CIoU loss is shown in formulas 10–12.


$$L_{\text{CIoU}}=1-\text{IoU}+\frac{{\text{ρ}}^{2}\left(\text{x},{\text{y}}_{\text{gt}}\right)}{{\text{C}}^{2}}+\text{αυ}$$ (10)

$$\text{υ}=\frac{4}{\text{π}}{\left(\text{arc}\tan\frac{{\text{ω}}_{\text{gt}}}{{\text{h}}_{\text{gt}}}\text{arc}\tan\frac{\text{ω}}{\text{h}}\right)}^{2}$$ (11)

$$\text{α}=\frac{\text{υ}}{\left(1-\text{IoU}\right)+\text{υ}}$$ (12)

In the formula, x represents the centroid of the predicted box, while y_gt_ denotes the centroid of the ground truth box. C represents the diagonal distance of the smallest area that encompasses both the predicted and actual boxes. The ρ denotes the Euclidean distance between the centroids of the predicted and ground truth boxes. Additionally, α signifies the weight coefficient, while v reflects the difference in aspect ratio between the predicted and actual boxes. By calculating the derivatives of ω and h, the corresponding gradients can be derived, as illustrated in formulas 13–15.


$$\frac{\partial \upsilon }{\partial \omega }=\frac{4}{\pi^{2}}{\left(arc\tan\frac{\omega_{gt}}{h_{gt}}arc\tan\frac{\omega }{h}\right)}^{2}\times \frac{h}{\omega^{2}+h^{2}}$$ (13)

$$\frac{\partial \upsilon }{\partial h}=-\frac{4}{\pi^{2}}{\left(arc\tan\frac{\omega_{gt}}{h_{gt}}arc\tan\frac{\omega }{h}\right)}^{2}\times \frac{\omega }{\omega^{2}+h^{2}}$$ (14)

$$\frac{\partial \upsilon }{\partial \omega }=-\frac{h}{\omega }\frac{\partial \upsilon }{\partial h}$$ (15)

The gradient values of ω and h are inversely proportional, meaning that these two parameters cannot be adjusted simultaneously during training, as doing so may negatively affect the training performance. Additionally, a limitation of CIoU Loss is that it neglects the directional alignment between the predicted and ground truth boxes, which can reduce network efficiency, slow convergence, and lead to inaccurate regression results. To address these issues, this experiment introduces the Focal-EIoU loss function, which combines Focal Loss and EIoU Loss methods, and incorporates an attention mechanism. By directing attention to different feature layers, the model’s focus on key areas is enhanced, improving both the accuracy and efficiency of detection. The formulas for the Focal EI-oU loss function are shown in 16 and 17


$${\text{L}}_{\text{Focal-EIoU}}{\text{=IoU}}^{\text{γ}}{\text{L}}_{\text{EIoU}}$$ (16)

$${\text{L}}_{\text{EIoU}}{\text{=L}}_{\text{IoU}}{\text{+L}}_{\text{dis}}{\text{+L}}_{\text{asp}}\text{=1-IoU+}\frac{{\text{P}}^{\text{2}}{\text{(x,y}}_{\text{gt}}\text{)}}{{{\text{(ω}}_{\text{c}}\text{)}}^{\text{2}}{\text{+(h}}_{\text{c}}{\text{)}}^{\text{2}}}+\frac{{\text{ρ}}^{\text{2}}{\text{(ω,ω}}_{\text{gt}}\text{)}}{{{\text{(ω}}_{\text{c}}\text{)}}^{\text{2}}}+\frac{{\text{ρ}}^{\text{2}}{\text{(h,h}}_{\text{gt}}\text{)}}{{{\text{(h}}_{\text{c}}\text{)}}^{\text{2}}}$$ (17)

Here, γ is set to 0.5, while ω_c_ and h_c_ represent the breadth and height of the minimum bounding rectangle that contains both the predicted and ground truth boxes. The Euclidean distance between the two center points is denoted by ρ. Additionally, L_IOU_, L_dis_, and L_asp_ correspond to the IoU loss, distance loss, and aspect ratio loss in the EIoU Loss, respectively.

### 3.3. Experimental environment

The experiment was conducted from September 2023 to July 2024 at the Oasis Laboratory of Tarim University. The experimental environment configuration is detailed in Table 4. The operating system employed was Windows 10 Professional, running on an Intel Xeon W-2223 processor. Additionally, the system was equipped with an NVIDIA RTX 3080 graphics card. The CUDA version was 11.7, and the Pytorch 1.10.0 framework was employed alongside Python 3.8.0. The stochastic gradient descent (SGD) optimization method was utilized during training, featuring an initial learning rate of 0.01, a momentum of 0.937, a weight decay coefficient of 0.0005, and a batch size of 16. The training process spanned a total of 300 epochs. Detailed configurations of the experimental parameters can be found in Table 4.

**Table 4:** Experimental parameter configuration.

Number	Experimental Description	Default Parameters
1	Number of epochs	300
2	Batch size	16
3	Image size	640×640 pixels
4	Concurrent Threads	16
5	Optimizer	AdamW
6	Initial Learning rate	0.01
7	Momentum	0.937
8	Weight decay coefficient	0.0005

### 3.4. Evaluation metrics

This study employed a variety of evaluation metrics to assess the model’s performance, including precision, recall, mAP_@0.5_ (mean average precision at an IoU threshold of 0.5), accuracy, F1-Score, and model size. The corresponding formulas are provided in Equations 18–23.


$$\text{Precision}=\frac{\text{True Positive}}{\text{True positive}+\text{False Positive}}$$ (18)

$$\text{Recall}=\frac{\text{True Positive}}{\text{True positive}+\text{False Negative}}$$ (19)

$$\text{Average Precision}=\overset{1}{\underset{0}{\int }}\text{Percision}\left(\text{Recall}\right)\text{d}\left(\text{Recall}\right)$$ (20)

$$\text{Mean Average Precision}=\frac{\sum_{1}^{M}\int_{0}^{1}\text{Percision}\left(\text{Recall}\right)\text{d}\left(\text{Recall}\right)}{M}$$ (21)

$$Accuracy=\frac{Numberofcorrectlypredictedsamples}{Totalnumberofsamples}$$ (22)

$$F1-Score=2\frac{Percision\times Recall}{Percision+Recall}$$ (23)

PSNR (Peak Signal-to-Noise Ratio) [51,52] is a metric used to quantify the difference between a reconstructed image and its original counterpart. It assesses image quality by calculating the ratio of the highest achievable signal power to the noise power in the image, expressed in decibels, as shown in Equation 24. In this context, MAX_I_ represents the maximum possible pixel value in the image (for example, 255 for an 8-bit image), while MSE (mean square error) refers to the mean of the squared differences between the reconstructed and original images. A higher PSNR value indicates a smaller difference between the images, signifying better image quality. Generally, a PSNR value greater than 30 dB is considered indicative of good image quality.


$$PSNR=10\times log_{10}\left(\frac{MAX_{I}^{2}}{MSE}\right)$$ (24)

SSIM (Structural Similarity Index Measure) [53,54] is a measure employed to assess the resemblance between two images in terms of structure, brightness, and contrast. It prioritizes the human eye’s perception of image quality, making it more aligned with the characteristics of the human visual system compared to PSNR. An SSIM value closer to 1 indicates higher similarity between the images and better quality. The formula is provided in Equation 25, where μ_1_ and μ_2_ represent the average brightness of the image, $\sigma_{x}^{2}$and $\sigma_{y}^{2}$ denote the image variance, $\sigma_{xy}$ is the covariance of the two images, and C_1_ and C_2_ are constants used to stabilize the denominator.


$$\text{SSIM}\left(\text{x},\text{y}\right)=\frac{\left(2{\text{μ}}_{\text{x}}{\text{μ}}_{\text{y}}+{\text{C}}_{1}\right)\left(2{\text{σ}}_{\text{xy}}+{\text{C}}_{2}\right)}{\left({\text{μ}}_{\text{x}}^{2}+{\text{μ}}_{\text{y}}^{2}+{\text{C}}_{1}\right)\left({\text{σ}}_{\text{x}}^{2}+{\text{σ}}_{\text{y}}^{2}+{\text{C}}_{2}\right)}$$ (25)

FID (Fréchet Inception Distance), introduced by Heusel et al. [55,56], extracts high-level features from images using a pre-trained Inceptionv3 model. It compares the distribution differences between the synthesized image and the actual image within these feature spaces. A smaller FID value indicates that the distribution of the synthesized image is more similar to that of the real image, reflecting higher quality. The formula is provided in Equation 26.


$$\text{FID}=∥{\text{μ}}_{\text{r}}-{\text{μ}}_{\text{g}}{∥}_{2}^{2}-{\text{T}}_{\text{r}}\left({\text{Σ}}_{\text{r}}-{\text{Σ}}_{\text{g}}-2{\left({\text{Σ}}_{\text{r}}{\text{Σ}}_{\text{g}}\right)}^{2}\right)$$ (26)

μ_r_ and μ_g_ denote the features extracted from the real image and the generated image, respectively. Σ_r_ and Σ_g_ represent the feature covariance matrices extracted from the real image and the generated image, respectively. d denotes the Euclidean distance between the two mean vectors, while Tr(Σ) indicates the trace of the covariance matrix.

## 4. Results

### 4.1. Experimental results of pseudo-images of apple leaf diseases generated by CycleGAN

**Fig 11:**
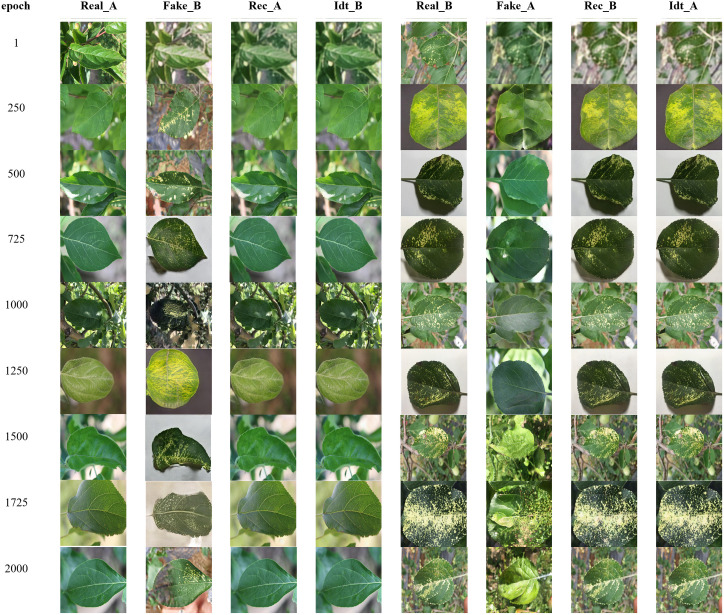
Using CycleGAN to train the apple mosaic disease process.

In the study of apple leaf disease detection, generating high-quality pseudo-defect images is a crucial task. The CycleGAN model can transform healthy leaf images into diseased images while preserving the background and color of the original image by performing image conversion between two domains (domain A and domain B). As illustrated in Fig 11, the generation of pseudo-defect images for apple mosaic disease serves as an example; the model was trained for 2000 iterations, and the conversion effects at each stage of training were meticulously recorded. I During CycleGAN training, Real_A and Real_B represent the real data from source domain A and target domain B, respectively, while Fake_B and Fake_A denote the fake data generated by the model. Additionally, Rec_A and Rec_B refer to the original data reconstructed from the generated data, and Idt_A and Idt_B represent the outputs that maintain the identity of the input data unchanged.

During the training process, the quality of the generated pseudo-defect images gradually improved with the increasing number of iterations. At 250 iterations, the initial characteristics of the disease were observable, although the tone and clarity remained insufficient. By 500 iterations, the image quality enhanced, and the disease characteristics became more pronounced. After 1000 iterations, the generated images closely resembled the real ones, with normalized tones, making the reconstructed images nearly indistinguishable from the originals. Finally, around 1725 iterations, the model achieved its optimal state, producing highly realistic and consistent pseudo-defect images for both diseased and healthy leaves.

To evaluate the quality of sample images generated by the improved CycleGAN-M network, comparative experiments were designed and conducted, with the results presented in Table 5 From the table, it can be concluded that the PSNR of Alternaria leaf spot generated by the improved CycleGAN-M network increased by 0.57 dB, the SSIM improved by 0.0529, and the FID decreased by 0.82. For Brown spot leaves, the PSNR improved by 1.45 dB, the SSIM increased by 0.0516, and the FID decreased by 1.34. The PSNR of the Frogeye leaf spot improved significantly by 7.72 dB, with the SSIM increasing by 0.2041 and the FID decreasing by 1.50. The PSNR for Mosaic leaf improved by 9.62 dB, with the SSIM rising by 0.3086 and the FID decreasing by 1.97. The PSNR of Powdery mildew leaves increased by 3.02 dB, with the SSIM improving by 0.1071 and the FID decreasing by 0.86. For Rust leaves, the PSNR improved by 0.78 dB, the SSIM increased by 0.1004, and the FID decreased by 2.09. Finally, the PSNR of the generated Scab leaves increased by 0.72 dB, the SSIM rose by 0.0421, and the FID decreased by 2.32. These experimental results demonstrate that the improved CycleGAN-M can more effectively reconstruct disease images that closely resemble real ones when processing various types of apple leaf diseases.

**Table 5:** The values of PNSR, SSIM, and FID for the data generated by CycleGAN.

Number	Model	PNSR/dB	SSIM	FID
Alternaria leaf spot	CycleGAN	26.16	0.7191	35.73
	CycleGAN-M	26.73	0.7720	34.91
Brown spot	CycleGAN	23.07	0.6938	51.90
	CycleGAN-M	24.52	0.7454	50.56
Frogeye leaf spot	CycleGAN	21.06	0.5264	32.69
	CycleGAN-M	28.78	0.7305	31.19
Mosaic	CycleGAN	18.71	0.5264	40.50
	CycleGAN-M	28.33	0.8350	38.53
Powdery mildew	CycleGAN	26.23	0.5969	58.99
	CycleGAN-M	29.25	0.7040	58.13
Rust	CycleGAN	25.66	0.6560	68.47
	CycleGAN-M	26.44	0.7564	66.38
Scab	CycleGAN	31.35	0.8079	54.26
	CycleGAN-M	32.07	0.8500	51.94

To verify the effectiveness of the improved CycleGAN-M data enhancement method on model detection performance, this study conducted comparative experiments using the MobileNet, AlexNet, GoogLeNet, and YOLOv8s models under three dataset conditions: (1) utilizing half of the real dataset (denoted as Datasets A), (2) using the original dataset (denoted as Datasets B), and (3) adding pseudo-image data to the training set of all real datasets in a 6:4 ratio (denoted as Datasets C, where real images and pseudo images are mixed). The results, as shown in Table 6, indicate that training with the improved CycleGAN-M network to expand the dataset enhances both precision and recall rates compared to the original dataset. This experiment demonstrates that the CycleGAN-M data enhancement method can significantly improve the accuracy of apple leaf disease image recognition and has broad applicability.

**Table 6:** Test experiments on dataset enhancement on different models.

Measuring model	Datasets A		Datasets B		Datasets C	
	Precision (%)	Recall (%)	Precision (%)	Recall (%)	Precision (%)	Recall (%)
MobileNet	60.0	69.0	72.0	72.0	74.0	74.0
AlexNet	77.7	77.3	80.2	80.5	81.8	82.0
GoogleNet	71.5	71.1	74.2	74.5	74.5	75.3
VGGNet	83.9	83.1	84.5	84.2	84.5	84.6
YOLOv8s	85.2	77.7	84.0	89.6	87.8	91.3

### 4.2. Experimental results

Table 7 presents the evaluation results of the YOLOv8s-KEF model compared to the original YOLOv8s model across several performance indicators, including precision, recall, mAP_@0.5_, and F1-Score. The YOLOv8s-KEF model demonstrated significant improvements in all evaluated metrics. On the training set, precision increased from 89.5% to 95.0%, recall rose from 91.4% to 93.1%, mAP_@0.5_ improved from 93.1% to 96.1%, and F1-Score increased from 90.4% to 94.5%. On the validation set, precision improved from 89.7% to 92.8%, recall increased from 88.9% to 92.3%, mAP_@0.5_ rose from 93.4% to 94.8%, and F1-Score enhanced from 89.1% to 92.5%. These experimental results indicate that the improved YOLOv8s-KEF model not only achieves higher detection accuracy and stability on the training set but also demonstrates excellent generalization and robustness on the validation set. Overall, the performance of the YOLOv8s-KEF model significantly surpasses that of the original YOLOv8s model across various performance indices.

**Table 7:** Comparison of model performance before and after improvement.

Model	Dataset Division	Precision (%)	Recall (%)	mAP_@0.5_ (%)	F1-Score (%)
YOLOv8s	Train	89.5	91.4	93.1	90.4
	Validation	89.7	88.9	93.4	89,1
YOLOv8s-KEF	Train	95.0	93.1	96.1	94.5
	Validation	92.8	92.3	94.8	92.5

**Fig 12:**
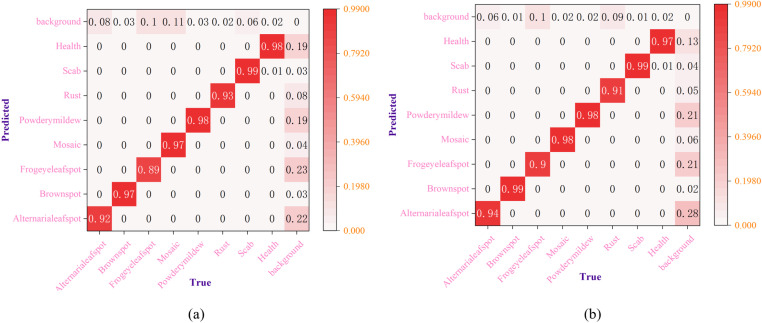
Normalized confusion matrix of the model before and after improvement: (a) YOLOv8s; (b) YOLOV8s-KEF.

Fig 12 presents the normalized confusion matrix for the detection of various apple leaf diseases using both the original YOLOv8s model and the enhanced YOLOv8s-KEF model. The confusion matrix offers a straightforward and easy-to-understand comparison of the model’s performance in recognizing various apple leaf diseases, both before and after the enhancements. The accuracy for the improved categories—Alternaria leaf spot, Brown spot, Frogeye leaf spot, and Mosaic—rose from 92.0%, 97.0%, 89.0%, and 97.0% to 94.0%, 99.0%, 90.0%, and 98.0%, respectively. Additionally, the misclassification rates for Alternaria leaf spot, Brown spot, Mosaic, and Scab predicted backgrounds decreased from 8.0%, 3.0%, 11.0%, and 6.0% to 6.0%, 1.0%, 2.0%, and 1.0%, respectively, further enhancing the models’ ability to differentiate these categories from the background. These experimental results demonstrate that the improved YOLOv8s-KEF model significantly enhances accuracy across multiple categories, particularly by reducing background misclassification rates and improving the distinction between complex categories.

**Fig 13:**
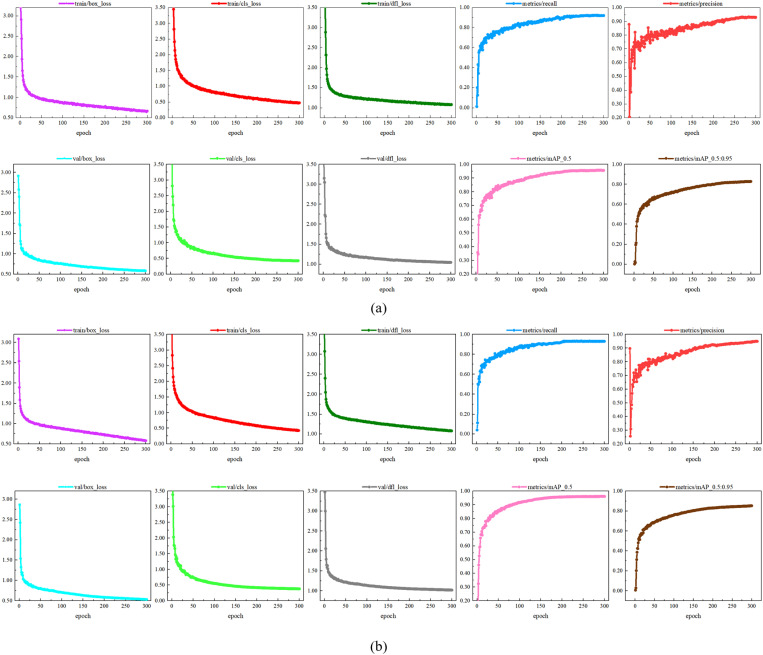
Model training results before and after improvement: (a) YOLOv8s (b) YOLOV8s-KEF.

Fig 13 illustrates the training results for both the original YOLOv8s model and the improved YOLOv8s-KEF model. In terms of loss function convergence, the box loss for both models demonstrates a positive downward trend, ultimately stabilizing at a lower value. However, the classification loss of the YOLOv8s-KEF model decreases more rapidly and converges at a lower point, indicating superior performance in object category prediction. The DFL loss of the YOLOv8s-KEF model also converges slightly faster than that of the original YOLOv8s model, suggesting enhanced accuracy in predicting object boundaries. Regarding precision and recall, both models achieve precision levels close to 90.0%, but the YOLOv8s-KEF exhibits greater stability with less fluctuation. The recall rate is similarly around 90.0%, with the YOLOv8s-KEF showing a slightly faster increase and better early-stage performance. In terms of mAP (mean Average Precision), the YOLOv8s-KEF model improves earlier and ends slightly higher, particularly in detection performance under the 95% IoU threshold. These experimental results demonstrate that our model outperforms YOLOv8s in convergence speed, classification accuracy, and detection performance. The YOLOv8s-KEF model reaches a stable state more quickly, and its training curve is smoother, indicating that its optimization strategy is more effective.

**Fig 14:**
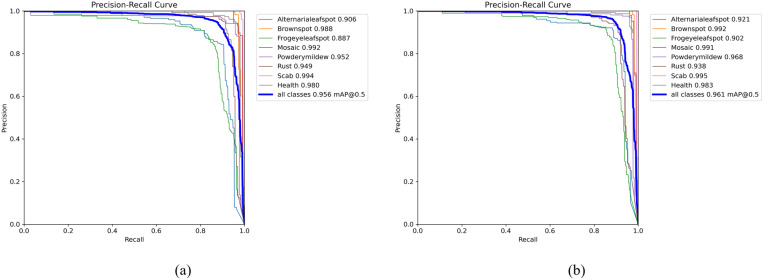
The precision-recall curves before and after the improvement are analyzed: (a) YOLOv8s; (b) YOLOv8s-KEF.

The precision-recall curve illustrates the trade-off between precision and recall across different thresholds. As precision increases, the false positive rate decreases, whereas an increase in recall corresponds to a decrease in the false negative rate. A larger area under the curve indicates superior model performance in terms of both precision and recall. Fig 14 demonstrates that the YOLOv8s model achieves an average precision (mAP) of 0.956, while the improved YOLOv8s-KEF model attains a mAP of 0.961 on the same curve, indicating enhanced performance.

**Fig 15:**
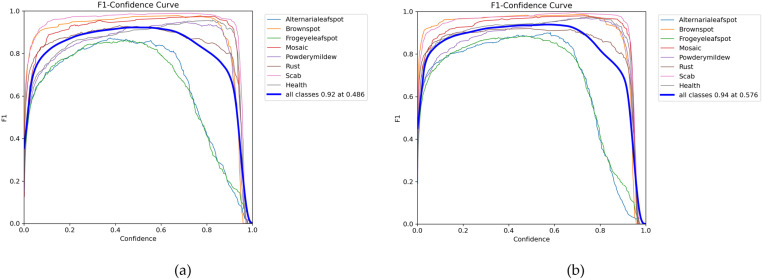
The F1 curves before and after improvement are compared: (a) YOLOv8s; (b) YOLOv8s-KEF.

The F1 metric is a crucial measure for evaluating classifier performance, as it balances the contributions of precision (P) and recall (R) by calculating their harmonic mean to derive the F1 score. Fig 15 illustrates the comparison of F1 curves before and after the improvement. In the original YOLOv8s model, the highest F1 score of 0.92 is achieved at a confidence value of 0.486. In contrast, the improved YOLOv8s-KEF model exhibits an increased confidence value of 0.576, resulting in a maximum F1 score of 0.94. This outcome indicates that the YOLOv8s-KEF model demonstrates significant enhancements in both confidence and F1 score, reflecting a better balance between precision and recall.

**Fig 16:**
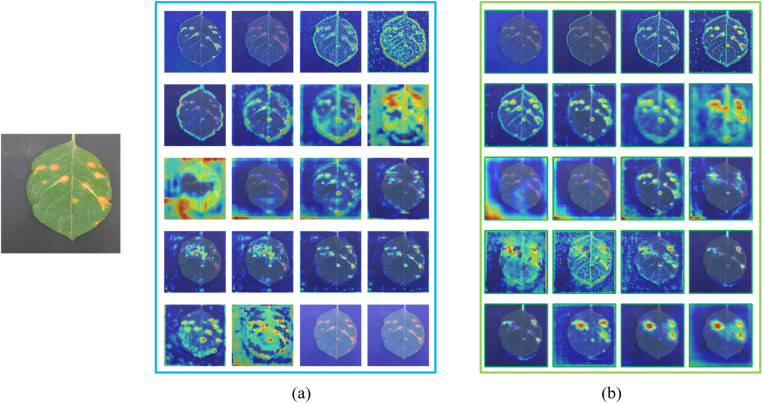
GradCAM **++** **visualizations of various network layers before and after model improvements:** (a)YOLOv8s; (b) YOLOv8s-KEF.

Fig 16 illustrates the Grad-CAM++ visualizations of the model before and after improvement across different network layers. The experimental results indicate that YOLOv8s-KEF exhibits a stronger capacity for extracting disease features in the Grad-CAM++ graphs at various network layers, particularly in mid- and high-level feature representations, compared to YOLOv8s. This suggests that the enhanced YOLOv8s-KEF network is more effective at capturing detailed information about the disease, thereby improving the model’s performance in disease detection tasks.

**Fig 17:**
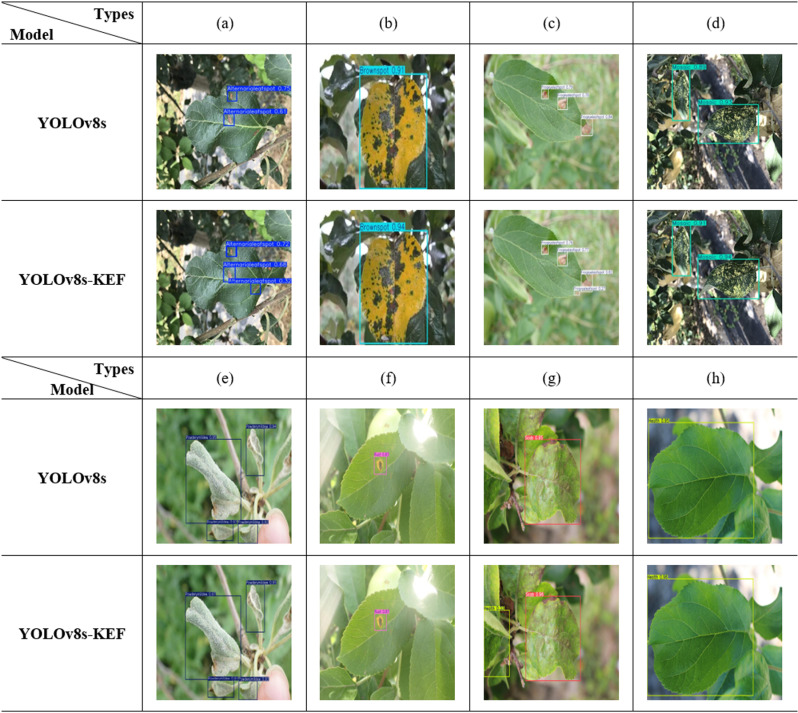
Comparison of model detection results before and after improvement: (a) Alternaria leaf spot; (b) Brown spot; (c) Frogeye leaf spot; (d) Mosaic; (e) Powdery mildew; (f) Rust; (g) Scab; (h) Health.

Fig 17 presents the visual results of apple leaf disease detection. Compared to the basic YOLOv8s model, the improved YOLOv8s-KEF model exhibits higher detection confidence, successfully identifies smaller lesion targets, and significantly reduces missed detections. These enhancements not only improve detection accuracy and reliability but also bolster the model’s ability to identify edge diseases in apple leaves. The experimental results clearly demonstrate that the YOLOv8s-KEF model performs better in practical applications and is more effective in handling the task of apple leaf disease detection.

### 4.3. Ablation experiment

Valuable insights into the interactions of various components within the object detection algorithm are provided by ablation experiments, enabling the optimization of model parameters and enhancing overall performance. As shown in Table 8, this ablation experiment gradually introduced three modules: C2f-KANConv, EMSDHead, and the Focal-EIoU loss function for boundary regression, significantly improving the performance of the YOLOv8s model. The original YOLOv8s model achieved precision, recall, mAP_@0.5_, accuracy, and F1-Score values of 87.8%, 91.3%, 85.4%, 89.3%, and 89.5%, respectively. After incorporating the C2f-KANConv module, accuracy and mAP_@0.5_ increased by 4.1% and 9.1%, respectively. The addition of the EMSDHead module improved precision, recall, mAP_@0.5_, and F1-Score by 5.6%, 2.8%, 10.5%, and 3.8%, respectively. When employing the Focal-EIoU loss function for boundary regression, accuracy, mAP_@0.5_, and F1-Score increased by 3.7%, 8.6%, 2.4%, and 2.0%, respectively. Furthermore, compared to the original YOLOv8s model, the combination of the C2f-KANConv and EMSDHead modules resulted in improvements of 5.4% in accuracy, 10.4% in mAP_@0.5_, 3.7% in accuracy, and 3.3% in F1-Score. Simultaneously using the C2f-KANConv module and the Focal-EIoU loss function yielded increases of 5.7%, 9.7%, 2.7%, and 2.9% in accuracy, mAP_@0.5_, accuracy, and F1-Score, respectively. When adding the EMSDHead module along with the Focal-EIoU loss function, the accuracy, mAP_@0.5_, accuracy, and F1-Score improved by 7.0%, 10.6%, 4.2%, and 4.2%, respectively. Overall, our YOLOv8s-KEF model demonstrates improvements of 8.0% in precision, 1.8% in recall, 10.7% in mAP_@0.5_, 6.5% in accuracy, and 5.0% in F1-Score compared to the original YOLOv8s model. The results in Table 8 indicate that the improved YOLOv8s-KEF model is more advantageous in the task of apple leaf disease detection.

**Table 8:** Ablation experiment.

C2f_KANConv	PWDHead	Focal-EIoU	Precision (%)	Recall (%)	mAP_@0.5_ (%)	Accuracy (%)	F1-Score(%)
×	×	×	87.8	91.3	85.4	89.3	89.5
√	×	×	91.9	90.4	94.5	90.9	91.1
×	√	×	93.4	93.2	95.9	90.1	93.30
×	×	√	91.5	91.5	94.0	91.7	91.5
√	√	×	93.2	92.7	95.8	93.0	92.8
√	×	√	93.5	91.4	95.1	92.6	92.4
×	√	√	94.8	92.6	96.0	93.5	93.7
√	√	√	95.0	93.1	96.1	95.8	94.5

### 4.4. Experiments on model performance using different C2f improvement modules

Table 9 presents the detection results for the improved YOLOv8s models: C2f-Faster [57], C2f-ODConv [58], C2f-iRMB [59] and C2f-DCNV2 [60], which were obtained by substituting the standard convolution in C2f with FasterConv, ODConv, DSConv, RConv and DConv. The experimental findings indicate that the model utilizing C2f-KAN achieves an accuracy of 91.9%, representing a 13.8% increase compared to C2f-ODConv. Furthermore, the recall rate for C2f-KAN is 90.4%, which surpasses that of C2f-ODConv and C2f-Faster by 11.1% and 7.1%, respectively. When employing C2f-KAN, the mAP@0.5 reaches 94.5%, exceeding the value for C2f-iRMB by 5.1%. Additionally, the mAP@0.5 for C2f-ODConv stands at 84.6%, which is 8.2% higher than previously reported. Moreover, C2f-KAN achieves an F1-Score of 91.1%, indicating an improvement of 3.2% over C2f-DCNV2. These experimental results confirm the efficacy of C2f-KAN in enhancing model detection performance. Table 9 Performance comparison experiment of different C2f improved modules.

**Table 9:** Performance comparison experiment of different C2f improved modules.

Model	C2f Improvement	Precision (%)	Recall (%)	mAP_@0.5_ (%)	mAP_@0.5_0.95_ (%)	F1-Score(%)
YOLOv8s	C2f-Faster	76.4	83.3	85.8	75.4	79.7
	C2f-ODConv	78.1	83.5	86.3	76.4	80.6
	C2f-iRMB	80.4	87.4	89.4	79.2	83.7
	C2f-DCNV2	86.4	89.8	91.8	82.5	87.9
	C2f-KAN	91.9	90.4	94.5	84.6	91.1

### 4.5. Comparison of different loss functions

Table 10 presents the experimental results of various IoU loss functions applied to bounding box regression. The results indicate that the model utilizing the Focal-EIoU loss function achieves precision and recall rates of 91.5% each, with a mAP@0.5 of 94.0% and a mAP@0.5_0.95 of 84.4%. Additionally, the F1-Score for this configuration is also 91.5%. In contrast, the DIoU loss function yields a precision of 90.2% and a recall of 90.8%, both of which are lower than those achieved with Focal-EIoU. The mAP@0.5_0.95 values for the SIoU and Wise-IoU loss functions are 83.7% and 83.6%, respectively, which are again inferior to the performance of Focal-EIoU. These findings demonstrate that the Focal-EIoU loss function provides the best overall model performance, achieving the highest detection accuracy.

**Table 10:** The experimental results of different loss functions are compared.

Model	Loss Function	Precision (%)	Recall (%)	mAP@0.5 (%)	mAP@0.5_0.95 (%)	F1-Score(%)
YOLOv8s	DIoU	90.2	90.8	93.8	84.1	90.5
	EIoU	90.6	91.0	93.3	83.3	90.8
	SIoU	89.5	92.0	93.5	83.7	90.7
	MPDIoU	90.2	90.6	93.7	83.8	90.4
	Shape-IoU	89.5	90.7	93.2	83.5	90.1
	Wise-IoU	89.4	91.1	93.5	83.6	90.3
	Focal-IoU	89.9	91.1	93.9	84.2	90.6
	Focal-EIoU	91.5	91.5	94.0	84.4	91.5

### 4.6. Comparative experiments on different network models

**Fig 18:**
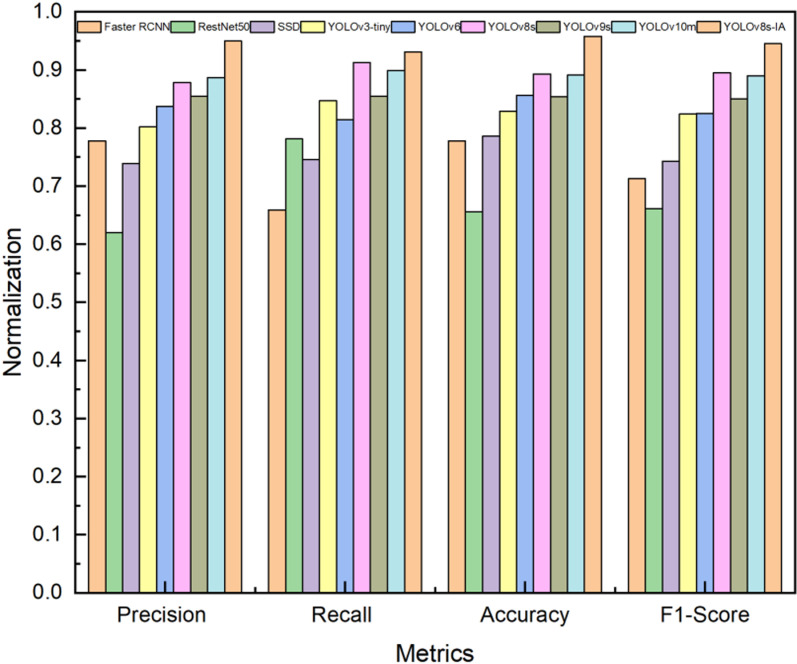
Normalization effect of performance indicators of different detection models for apple leaf diseases.

To comprehensively evaluate the performance of our proposed YOLOv8s-KEF model, we selected several state-of-the-art detection models as baselines, including Faster R-CNN [61], ResNet50 [62], SSD [63], YOLOv3-tiny [64], YOLOv6 [65], YOLOv9s [66] and YOLOv10m [67]. These models were chosen for their widespread use in plant disease detection tasks and their relevance to lightweight detection frameworks. As shown in Table 11, the experimental data reveal that our proposed model, YOLOv8s-KEF, exhibits superior performance across multiple indicators. Specifically, YOLOv8s-KEF achieves an accuracy of 95.0%, significantly surpassing the 87.8% accuracy of the YOLOv8s model and the 83.7% accuracy of the YOLOv6 model. It also demonstrates impressive recall, with a rate of 93.1%, exceeding the 91.3% recall of the YOLOv8s model and the 84.7% recall of the YOLOv3-tiny model. Furthermore, the accuracy of the YOLOv8s-KEF model reaches 95.8%, notably higher than the 77.8% accuracy of the Faster R-CNN model and the 85.4% accuracy of the YOLOv9s model. The F1-Score of YOLOv8s-KEF is 94.5%, indicating its effectiveness in predicting positive classes and its ability to accurately identify positive class samples while maintaining high precision, outperforming other models. Despite its enhanced performance, the YOLOv8s-KEF model has a size of 28.5 MB, which is slightly larger than the 22.5 MB of the YOLOv8s model but smaller than the 33.5 MB of YOLOv10m, reflecting an efficient balance between performance and model size. Fig 18 illustrates the normalized performance indicators of different detection models. The experimental results demonstrate that the YOLOv8s-KEF model excels not only in detection accuracy but also in achieving a favorable balance between model size and performance, highlighting its strong application potential.

**Table 11:** Performance comparison of apple leaf disease detection models.

Model	Image Size	Precision (%)	Recall (%)	Accuracy (%)	F1-Score	Weight Size (MB)
Faster RCNN	320×320	77.8	65.9	77.8	71.3	107
RestNet50	224 x 224	62.0	78.1	65.6	66.1	246
SSD	512×512	73.9	74.6	78.6	74.3	106
YOLOv3-tiny	640×640	80.2	84.7	82.9	82.4	24.4
YOLOv6	640×640	83.7	81.4	85.67	82.5	8.7
YOLOv8s	640×640	87.8	91.3	89.3	89.5	22.5
YOLOv9s	640×640	85.5	85.5	85.4	85.0	15.3
YOLOv10m	640×640	88.7	89.9	89.1	89.0	33.5
YOLOv8s-KEF	640×640	95.0	93.1	95.8	94.5	28.5

**Fig 19:**
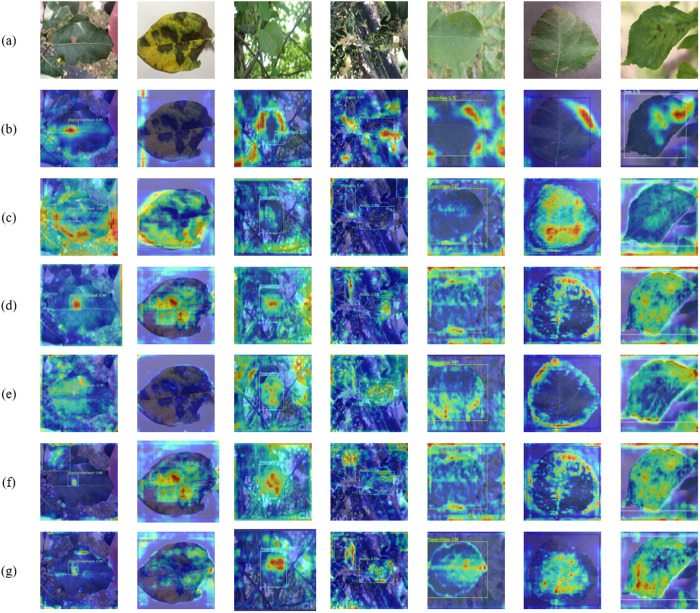
Visualization of different models of Grad-CAM on the test set: (a) original image; (b) YOLOv3-tiny; (c) YOLOv6; (d) YOLOv8s; (e) YOLOv9s; (f) YOLOv10m; (g) YOLOv8s-KEF. The images from left to right are Alternaria leaf spot, Brown spot, Frog eye leaf spot, Mosaic, Powdery mildew, Rust, and Scab.

To gain a deeper understanding of the attention and weight distribution of different models across various regions in the input image, Fig 19 presents the XGrad-CAM visualization analysis for YOLOv3-tiny, YOLOv6, YOLOv8s, YOLOv9s, YOLOv10m, and YOLOv8s-KEF in identifying different apple leaf diseases. These visualizations reveal the feature extraction capabilities of each model when addressing apple leaf diseases. Compared to the other models, YOLOv8s-KEF demonstrates a heightened focus on the diseased areas, accurately locating lesion features and exhibiting greater sensitivity in identifying minor lesions. The visualization results indicate that YOLOv8s-KEF excels in capturing disease features, particularly for minor diseases. In contrast to YOLOv9s and YOLOv10m, YOLOv8s-KEF has a more concentrated and clearer attention area. This precise attention capability enhances YOLOv8s-KEF’s reliability and practicality in real-world applications, providing essential support for the early detection and precise management of fruit tree diseases.

## 5. Conclusions

This study presents a deep learning-based network model for identifying apple leaf diseases, addressing challenges such as difficult dataset acquisition, insufficient sample size, and low recognition accuracy. Synthetic samples were generated using the CycleGAN-M network with a multi-scale attention mechanism, enhancing the model’s generalization and robustness. Additionally, the YOLOv8s-KEF model was improved by replacing the traditional C2f structure with C2f-KanConv and optimizing the detection head to implement efficient multi-scale convolution (EMS-Conv) and the Focal-EIoU loss function, significantly improving small target detection, overall accuracy, and detection precision.

Experimental results demonstrate notable improvements in precision, recall, accuracy, and F1-score, with precision increasing by 7.2% and accuracy by 6.5% compared to the original YOLOv8s model. The YOLOv8s-KEF model also outperforms mainstream detection models. By synthesizing complex diseased leaf data through CycleGAN and integrating it with the improved YOLOv8s-KEF model, this study provides innovative and effective solutions for fruit tree disease detection, offering substantial practical significance in promoting fruit tree health and increasing fruit yield.

## 6. Discussion

This study has made significant progress in apple leaf disease detection using the CycleGAN-M and YOLOv8s-KEF models, but there are still some limitations in complex environments, such as lighting changes, image noise, and overlapping or damaged leaves. Affects the detection effect of the model. In addition, the pseudo images generated by CycleGAN-M may not fully cover all disease scenarios in terms of quality and diversity, especially for rare diseases with limited datasets. Furthermore, the model’s high computational resource requirements limit its application in resource-limited environments such as small farms or mobile devices.

In the future, this study will adopt advanced data enhancement technologies such as 3D disease modeling or hyperspectral imaging technology to improve the adaptability of the model in complex scenarios. Improve the quality and diversity of fake images by using more powerful generative adversarial networks such as StyleGAN. The computing cost is reduced through lightweight model design, making it more suitable for edge devices. Expand the scope of application of the model to cover other crop disease detection and verify its cross-scenario performance.
